# The Chinese Herbal Medicine Formula *m*KG Suppresses Pulmonary Fibrosis of Mice Induced by Bleomycin

**DOI:** 10.3390/ijms17020238

**Published:** 2016-02-15

**Authors:** Ying Gao, Li-Fu Yao, Yang Zhao, Li-Man Wei, Peng Guo, Meng Yu, Bo Cao, Tan Li, Hong Chen, Zhong-Mei Zou

**Affiliations:** 1 Institute of Medicinal Plant Development, Chinese Academy of Medical Sciences and Peking Union Medical College, Beijing 100193, China; gying77@163.com (Y.G.); weilim13311035797@163.com (L.-M.W.); yumeng.5555@163.com (M.Y.); 2 Department of Pharmacy, Logistics College of Chinese People’s Armed Police Force, Tianjin 300309, China; leaf_yao@126.com (L.-F.Y.); guopengtj@163.com (P.G.); caobo19814@126.com (B.C.); tanli20042001@163.com (T.L.); 3 Tianjin University of Traditional Chinese Medicine, Tianjin 300193, China; youngzhao91@163.com

**Keywords:** pulmonary fibrosis, bleomycin, *m*KG (Modified Kushen Gancao Formula), mice

## Abstract

Pulmonary fibrosis (PF) is a serious progressive lung disease and it originates from inflammation-induced parenchymal injury with excessive extracellular matrix deposition to result in the destruction of the normal lung architecture. Modified Kushen Gancao Formula (*m*KG), derived from traditional Chinese herbal medicine, has a prominent anti-inflammatory effect. The present study is to explore the inhibitory effects of *m*KG on bleomycin (BLM)-induced pulmonary fibrosis in mice. *m*KG significantly decreased pulmonary alveolitis, fibrosis scores, and interleukin-6 (IL-6), interleukin-17 (IL-17), transforming growth factor-β (TGF-β) and hydroxyproline (HYP) levels in lung tissue of mice compared with BLM treatment. It markedly alleviated the increase of HYP content in the lung tissues and pathologic changes of pulmonary fibrosis caused by BLM instillation. In conclusion, *m*KG has an anti-fibrotic effect and might be employed as a therapeutic candidate agent for attenuating pulmonary fibrosis.

## 1. Introduction

Pulmonary fibrosis (PF) is a serious chronic lung disease with unknown pathogenesis. It is a progressive and lethal lung disease, characterized by the accumulation of extracellular matrix in the alveolar septum to result in the destruction of the normal lung architecture [1,2]. The current treatment for PF includes corticosteroids and immunosuppressive agents, but the outcomes of therapy are not satisfying. Traditional Chinese medicines (TCMs) are gaining more attention all over the world due to their holistic concept of TCM theory and long historical clinical practice. In Chinese herbal therapy, the most widely used medicines are combined by many herbs and prepared according to TCM formulation concepts.

We have optimized a three-herb TCM formula, *m*KG, derived from a famous traditional Chinese medicinal formula, Kushen Gancao Tang, which consists of the roots of *Angelica sinensis* (Oliv.) Diels. (Dang-gui), the roots of *Sophora flavescens* Ait. (Kushen) and the rhizome of *Glycyrrhiza uralensis* Fisch (Gancao). Kushen Gancao Tang has been recorded in *Zhong Miao Xian Fang*, a classic medical book published in the Ming dynasty of China. It is comprised of two medicinal herbs, Kushen and Gancao, which have been used to treat choleplania, infection, esoenteritis and dysentery for a long time. Now Kushen Gaocao Formula has been used in the clinic to treat hepatitis and immune system diseases [3]. In addition, it is reported to have anti-liver fibrosis activity [4]. Kushen has been used in China for its cleaning heat, drying humidity and diuresis for a long time. Pharmacological studies have shown its anti-angiogenic, anti-inflammatory and anti-nociceptive activities. So Kushen has been widely used for treating various inflammatory and infectious diseases in the clinic, such as eczema and upper respiratory tract infections. It has also been used for treating asthma by suppressing airway responses, decreasing inflammation and down-regulating Th2 levels in asthmatic-model mice [5]. Its active component oxymatrine can attenuate bleomycin-induced pulmonary fibrosis in mice by regulating the TGF-β/Smad signaling pathway [6,7]. Gancao has been used as an herbal medicine for centuries in China to treat various diseases such as gastric or duodenal ulcers, hepatitis, sore throats, coughs, bronchitis, arthritis, allergies, and cardiovascular disease [8,9]. Gancao and its active compounds including glycyrrhizin, isoliquiritigenin and β-glycyhrritinic acid have been shown to possess prominent anti-inflammatory activities and can inhibit reactive oxygen species (ROS) [10,11,12]. Glycyrrhizic acid can alleviate carbon tetrachloride-induced liver fibrosis and bleomycin-induced pulmonary fibrosis in rats [13,14]. Modern research showed that Dang-Gui has broad pharmacological effects on cardiovascular diseases, gynecological diseases and immune system disorders [15,16]. The combination of Dang-Gui and Kushen is extensively used in traditional Chinese medicine to treat inflammatory diseases, such as acne, heart disease, and hepatitis [17].

Considering the properties of those herbs, the Kushen Gancao Tang has been modified in expectation of the synergistic enhancement of its anti-inflammatory and anti-fibrotic effects. In addition, Modified Kushen Gancao Formula has been used to treat bronchial asthma and chronic obstructive pulmonary disease (COPD) in Pingjin Hospital, Tianjin, China, whereas there are no reports about the effects of Modified Kushen Gancao Formula on PF. Additionally, the mechanisms by which Modified Kushen Gancao Formula suppresses inflammatory and fibrotic effects have not been fully elucidated.

Although the mechanisms of PF are not understood fully, transforming growth factor-β (TGF-β) is recognized as a critical factor for inducing fibrosis. In the early period of PF, inflammatory factors such as interleukin-17 (IL-17) and interleukin-6 (IL-6) play an important role in autoimmunity. IL-17 is a novel family cytokine that consists of six protein members (from IL-17A to IL-17F), which play an important role in many chronic inflammatory diseases [18,19,20,21]. Bleomycin (BLM), an antineoplastic drug, has been reported to cause fibrosis in different species of animals [22,23,24]. The intratracheal instillation of BLM was widely accepted as the method to develop the model of lung fibrosis. Here we focused on exploring whether the *m*KG decreases the expression of TGF-β and inflammatory factors so that it can attenuate BLM-induced PF. In this study we evaluate the antifibrotic properties of *m*KG.

## 2. Results

### 2.1. Histological Analysis

**Figure 1:**
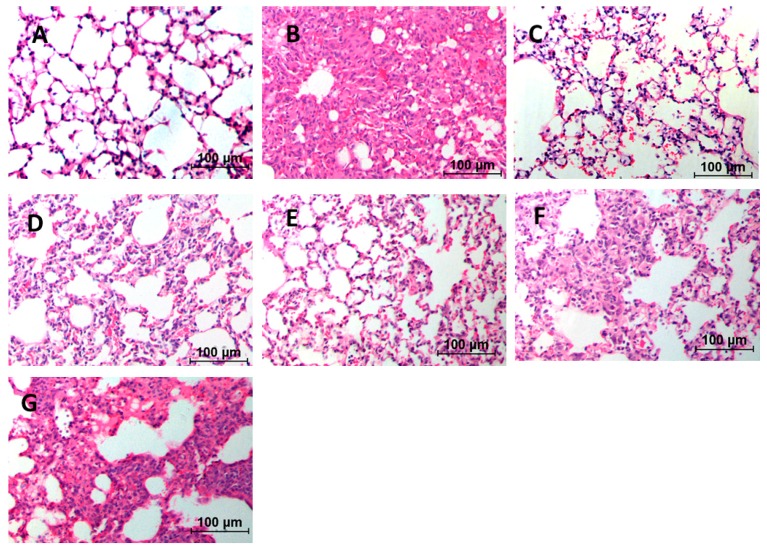
Effect of *m*KG on histopathologic changes in BLM-induced pulmonary fibrosis mice. (**A**) Normal group; (**B**) BLM group; (**C**) Pirfenidone group; (**D**) Dexamethasone group; (**E**) *m*KG-H group; (**F**) *m*KG-M group; (**G**) *m*KG-L group. All sections were stained with HE and representative sections are shown at the same magnification (200×).

**Figure 2:**
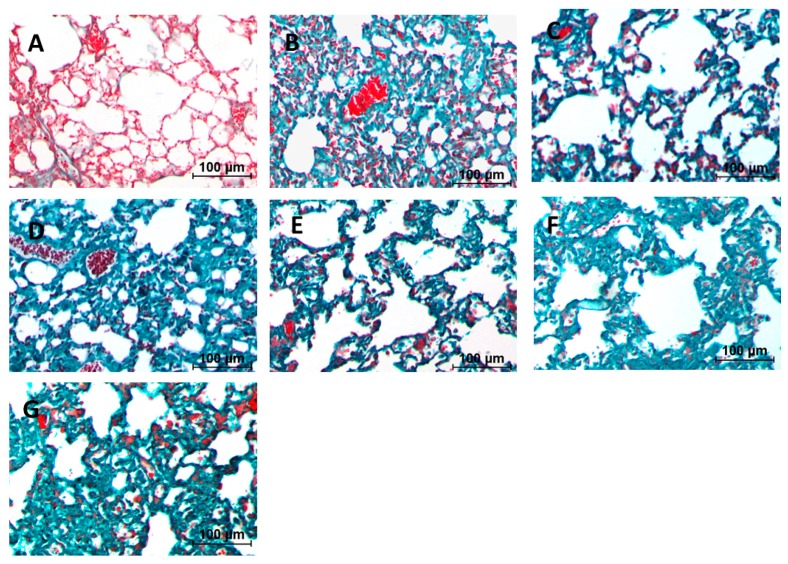
Effect of *m*KG on histopathologic changes in BLM-induced pulmonary fibrosis mice. (**A**) Normal group; (**B**) BLM group; (**C**) Pirfenidone group; (**D**) Dexamethasone group; (**E**) *m*KG-H group; (**F**) *m*KG-M group; (**G**) *m*KG-L group. All sections were stained with Masson trichrome and representative sections are shown at the same magnification (200×).

Numerous inflammatory cell infiltrations were observed in the alveolar and interstitial space of the lung by HE staining. From Figure 1 we can learn that mild edema became serious after inducing by BLM, with the structural confusion of the lung tissue and obvious swelling in the alveolar septum. Apparent alleviation of pulmonary alveolitis was observed in lung tissue of the pirfenidone group and high-dose *m*KG group, and the alveolar septum was thinner. Additionally, from the result of Masson staining (Figure 2), it showed that blue-stained collagen massively increased in the untreated group. Treatment with *m*KG alleviated the collagen deposit according to the result of the Masson staining. The effect was more significant when treated with pirfenidone and the high-dose Ganshen formula.

### 2.2. Expression of IL-6, IL-17 mRNAs in Lung Tissue

**Figure 3:**
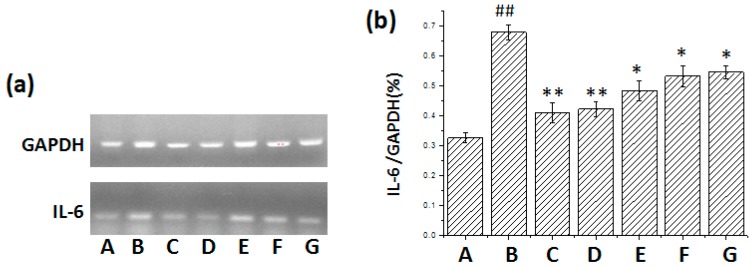
Effect of *m*KG on the mRNA Expression of IL-6 in the lung tissues of BLM-induced pulmonary fibrosis mice. (**a**) The mRNA expressions of IL-6 in the lung tissues were detected by RT-PCR; (**b**) The semiquantitative result of the IL-6 mRNA expression. Data were expressed as mean ± S.D. ^##^ *p* < 0.01 compared with the control group. * *p* < 0.05, ** *p* < 0.01 compared with the BLM group. (A) Normal group; (B) BLM group; (C) Pirfenidone group; (D) Dexamethasone group; (E) *m*KG-H group; (F) *m*KG-M group; (G) *m*KG-L group.

**Figure 4:**
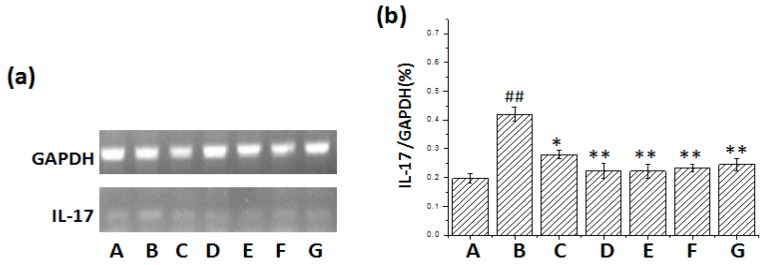
Effect of *m*KG on the mRNA Expression of IL-17 in the lung tissues of BLM-induced pulmonary fibrosis mice. (**a**) The mRNA expressions of IL-17 in the lung tissues were detected by RT-PCR; (**b**) The semiquantitative result of the IL-17 mRNA expression. Data were expressed as mean ± S.D. ^##^ *p* < 0.01 compared with the control group. * *p* < 0.05, ** *p* < 0.01 compared with the BLM group. (A) Normal group; (B) BLM group; (C) Pirfenidone group; (D) Dexamethasone group; (E) *m*KG-H group; (F) *m*KG-M group; (G) *m*KG-L group.

From Figure 3 and Figure 4 it was found that IL-6 and IL-17 mRNA expression increased in the lung tissue of mice after BLM administration. Treatment with pirfenidone and *m*KG attenuated IL-6 and IL-17 mRNA expression, and there was statistical significance with high-dose *m*KG.

### 2.3. Results of Immunohistochemistry Staining for Col-1 and Col-3

**Figure 5:**
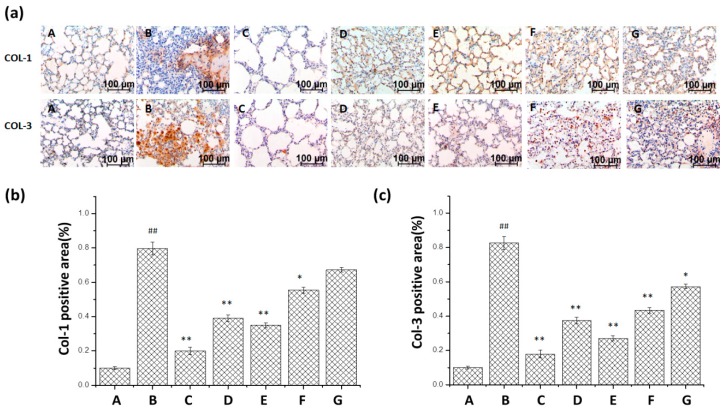
Effect of *m*KG on the mRNA Expression of Col-1, Col-3 in the lung tissues of BLM-induced pulmonary fibrosis mice by immunohistochemical analysis. (**a**) The Col-1, Col-3 expressions in the lung tissues were detected by immunohistochemical analysis (Col-1: upper panels; Col-3: lower panels). (Magnification, 200×); (**b**) The quantitative result of Col-1 expression; (**c**) The quantitative result of Col-3 expression. Data were expressed as mean ± S.D. ^##^ *p* < 0.01 compared with the control group. * *p* < 0.05, ** *p* < 0.01 compared with the BLM group. (A) Normal group; (B) BLM group; (C) Pirfenidone group; (D) Dexamethasone group; (E) *m*KG-H group; (F) *m*KG-M group; (G) *m*KG-L group.

In Figure 5, the expression of Col-1 and Col-3 was markedly increased in the BLM group when compared with the normal group, and the expression of Col-1 and Col-3 could be inhibited in groups that were treated with *m*KG, pirfenidone and dexamethasone by immunohistochemistry testing. Especially in the *m*KG-H group and positive control (pirfenidone and dexamethasone) groups, Col-1- and Col-3-stained area fractions were about half of that in the BLM group or even less.

### 2.4. Effect of mKG on Expression of TGF-*β*1, Hydroxyproline (HYP), IL-6 and IL-17

**Figure 6:**
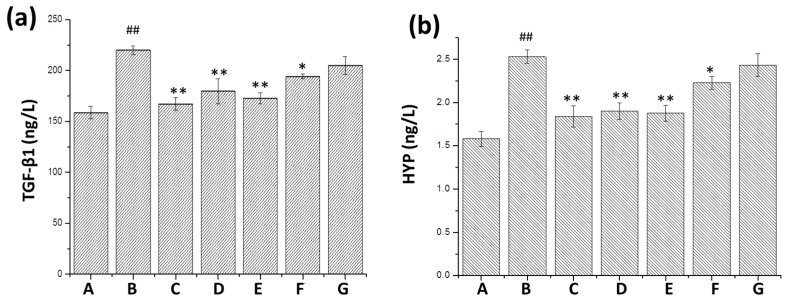

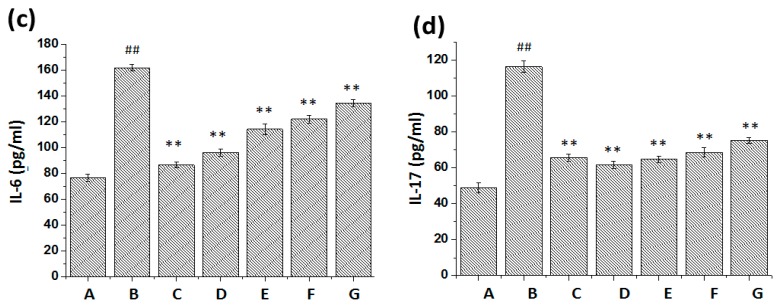
Effect of *m*KG on the content of TGF-β1, HYP, IL-6 and IL-17 in the lung tissues of BLM-induced pulmonary fibrosis mice by ELISA analysis. (**a**) TGF-β1 content in the lung tissues were detected by ELISA; (**b**) Hydroxyproline content in the lung tissues were detected by ELISA; (**c**) IL-6 content in the lung tissues were detected by ELISA; (**d**) IL-17 content in the lung tissues were detected by ELISA. Data were expressed as mean ± S.D. ^##^ *p* < 0.01 compared with the control group. * *p* < 0.05, ** *p* < 0.01 compared with the BLM group. (A) Normal group; (B) BLM group; (C) Pirfenidone group; (D) Dexamethasone group; (E) *m*KG-H group; (F) *m*KG-M group; (G) *m*KG-L group.

The result of ELISA showed that TGF-β1 and HYP levels were increased in the BLM group compared with the normal group, while they were decreased in the positive control group and *m*KG treatment group, especially in the high-dose *m*KG group, when compared with BLM group. IL-6 and IL-17 concentrations in the BLM group were obviously increased compared with the normal group, whereas the administration of *m*KG significantly reduced these two cytokines levels in the lung tissues (Figure 6).

### 2.5. Changes in Protein Expression of a-SMA

**Figure 7:**
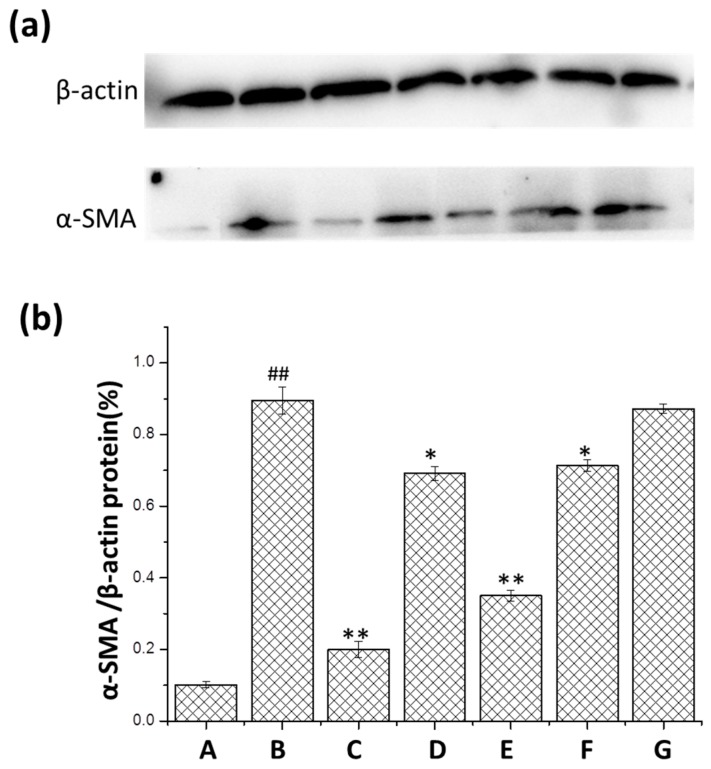
Effect of *m*KG on the expression of α-SMA in the lung tissues of BLM-induced pulmonary fibrosis mice by Western blot analysis. (**a**) The α-SMA expressions in the lung tissues were detected by Western blot analysis; (**b**) The quantitative result of the α-SMA expression. Data were expressed as mean ± S.D. ^##^ *p* < 0.01 compared with the control group. * *p* < 0.05, ** *p* < 0.01 compared with the BLM group. (A) Normal group; (B) BLM group; (C) Pirfenidone group; (D) Dexamethasone group; (E) *m*KG-H group; (F) *m*KG-M group; (G) *m*KG-L group.

The expression of α-SMA was tested by Western blot analysis (Figure 7). After modeling, α-SMA was obviously increased compared to the normal group. In the group treated with *m*KG, the expression of α-SMA was much lower than in the BLM group, especially in the high-dose group.

## 3. Discussion

PF is a chronic lung disease characterized by excessive accumulation of extracellular matrix (ECM) deposition to mesenchymal transition (EMT), which finally leads to the decline of lung function. Historically, pulmonary fibrosis was believed to result mainly from chronic inflammation [25]. The persistent immune response to injury led to inflammation, tissue remodeling and repair processes taking place constantly. Because environmental pollution is more and more serious, the incidence of PF has been on the rise in China recently, and it is a progressive and fatal pulmonary disease without proven drug therapies. Although the mechanisms of PF are not understood fully, transforming growth factor-β1 (TGF-β1) is recognized as a critical factor in inducing fibrosis [26,27,28]. Furthermore, studies have also confirmed that inflammation factors such as IL-6 and IL-17 contribute to the process of PF [29,30].

The pulmonary fibrosis model induced by BLM is widely used to study the anti-fibrotic effects of numerous drugs. BLM causes alveolar cell damage, inflammatory response, fibroblast proliferation and subsequent collagen content deposition [31,32]. This study was undertaken to demonstrate the antifibrotic properties of *m*KG. In our *in vivo* study, the body weight of rats decreased, the hydroxyproline content of the lungs increased, and pathologic changes of pulmonary fibrosis markedly appeared after BLM instillation. However, this situation got relieved in the *m*KG treatment groups. According to the results of the histological analysis, we can prove the inhibition effect of *m*KG on inflammation and pulmonary fibrosis. The upregulation of TGF-β1 expression is a consistent feature of most fibrotic diseases. Many studies have created a strong rationale for an antifibrotic strategy in which the principal objective of treatment is blocking TGF-β1. The inhibition effect of *m*KG on TGF-β1 could be shown from the results of the ELISA analysis and the expression of α-SMA was downregulated according to the results of the Western blot. Additionally, the results of the analysis of IL-6, IL-17A mRNA expression and the contents of these two cytokines in the lung tissues confirmed the anti-inflammatory effect of *m*KG.

PF is characterized by the remodeling of lung tissue and over-deposition of ECM [33,34]. Our previous studies suggested that *m*KG attenuated BLM-induced PF in rats via the inhibition of ECM. A main pathological cause of PF is the imbalance between the synthesis and degradation of ECM which increases the production of type I and type III collagen (Col-1, Col-3) [35]. Compared with the model group, *m*KG significantly reduced the content of HYP, Col-1 and Col-3, especially for the high-dose group. The α-smooth muscle actin (α-SMA) is an important downstream protein of TGF-β1. Myofibroblasts are highly synthetic for collagen, featured by the presence of α-SMAs, which are the primary effector cells during the progression of fibrosis [36,37]. The downregulation of the expression of α-SMA also confirmed the fact that *m*KG could inhibit the production of ECM.

## 4. Experimental Section

### 4.1. Reagents and Antibodies

All raw herbs were purchased from Beijing Tongren Tang Pharmaceutical Co., Ltd. (Beijing, China) and identified as the roots of *Angelica sinensis* (Oliv.) Diels., the roots of *Sophora flavescens* Ait., the rhizome of *Glycyrrhiza uralensis* Fisch. by Professor Yulin Lin of the Institute of Medicinal Plant Development (IMPLAD), Chinese Academy of Medical Sciences and Peking Union Medical College. The voucher specimen is deposited in our laboratory of IMPLAD.

Prestained protein marker was purchased from New England Biolabs (Beijing, China). Anti-albumin (Alb) (ab8940) sheep antibody, anti-a-smooth muscle actin (a-SMA) (ab5694) rabbit antibody were purchased from Abcam (Cambridge, MA, USA) and prepared in 1:3000, and 1:2000 dilution, respectively. The BCA Protein Assay Kit and the SuperSignal West Pico Chemiluminescent Substrate (ECL) were obtained from Pierce Chemical Company (Rockford, IL, USA). Trizol was obtained from Invitrogen (Carlsbad, CA, USA). The First-Strand cDNA Synthesis Kit (K1622) was purchased from Fermentas (St Leon-Roth, Germany). ELISA kits were obtained from Nanjing Jiancheng Bioengineering Institute (Nanjing, China).

### 4.2. Animals

A total of 70 BALB/c healthy male mice, weighing 20–22 g, were obtained from the Academy of Military Medical Experimental Animal Center. The mice were kept in community cages with 12 h periods of light and dark cycles and maintained on standard rodent chow with access to water *ad libitum*. All animal care and experimentation was approved by the principles and guidelines of the National Institutes of Health Guide for the Care and Use of Laboratory Animals.

### 4.3. Extract of mKS Preparation

Modified Kushen Gancao Formula (*m*KS) consists of Keushen Gancao Tang and Angelica oil. The *m*KS extract was prepared in our laboratory. Briefly, Gancao (the rhizome of *Glycyrrhiza uralensis*) 60 g and Kushen (the roots of *Sophora flavescens*) 60 g were soaked together in 1200 mL of 60% ethanol for 1 h at room temperature and thereafter refluxed for 1 h. The filtrate was collected and the residues were then refluxed twice in 1200 mL of 60% ethanol for 1 h. The three filtrates were combined and concentrated under vacuum to give the extract of Keushen Gancao Tang (yield 28.5%). Dang-gui (the roots of *Angelica sinensis*) 160 g was subjected to extraction separately in 1600 mL of water by the steam generator for 8 h at room temperature to extract the Angelica oil. The oil is collected, dried with anhydrous sodium sulphate and stored at 4 °C until use (yield 0.4%). Before the experiments, Angelica oil was added to the dry powder of Kushen Gancao Tang. Quality control of the final *m*KG extract was performed using UPLC-MS/MS and GC-MS (Supplementary Materials). Samples of *m*KG are available from the authors.

### 4.4. Groups of Animals and Treatments

Seventy BALB/c mice were randomly divided into seven groups (*n* = 10 per group): Normal control group, BLM-treated group, pirfenidone group, dexamethasone group and three *m*KG groups (*m*KG-L, *m*KG-M, *m*KG-H) at the dose of 0.41, 1.64 and 6.56 g/kg/day, respectively. Bleomycin was dissolved by sterile normal saline, and then mice in groups except in the normal control group were intratracheally injected with prepared bleomycin at a dose of 5 mg/kg. The normal control group was given equal amounts of normal saline. From the second day to the 21st day after modeling, mice in *m*KG groups or pirfenidone group were intragastrically administrated with prepared different dose *m*KG solution or prednisone (50 mg/kg), while mice in dexamethasone group were intraperitoneally injected with dexamethasone from the second day to the fourth day. Mice in normal control and BLM-treated group received equal amounts of normal saline. The body weight of mice was monitored every day. On the 21st day, the blood of the mice was drawn from the abdominal aorta under pentobarbital anesthesia and the lungs were removed. The left lungs were used for histological examination, and the right lungs were frozen in liquid nitrogen.

### 4.5. Histological Examination

The samples of left lung were fixed in 10% neutral formalin, embedded in paraffin and sectioned. The sections were stained with hematoxylin and eosin (HE) and Massons’s trichrome according to conventional methods. The slides were evaluated under a light microscope.

### 4.6. Measurement of Content of Hydroxyproline,TGF-β1, IL-6 and IL-17 in Lung Tissues

Hydroxyproline (HYP) content of lung tissue was measured by using hydroxyproline kits (Nanjing Jiancheng Biochemical Institute, Nanjing, China). TGF-β1 in lung tissue was tested by ELISA kit (Nanjing Jiancheng Biochemical Institute, Nanjing, China). IL-6 and IL-17 were tested by ELISA kit (Nanjing Jiancheng Biochemical Institute, Nanjing, China). It was performed according to the instruction of the manufacturer.

### 4.7. Immunohistochemistry Analysis

Paraffin-embedded tissue sections were rehydrated in xylene and graded ethanol solutions. The slides were exposed to methanol-hydrogen peroxide for 10 min [19], washed in phosphate buffered saline (PBS), and then antigen retrieval was made. After blocking with serum for 20 min, sections were then immunostained with type 1 collagen (Col-1) and type 3 collagen (Col-3) primary antibodies and incubated overnight at 4 °C. After washing the slides thrice with PBS, the sections were then incubated with secondary antibody for 20 min at room temperature. Sections were then washed with PBS and incubated with streptavidin-biotin-peroxidase complex for 10 min. After washing with PBS, diaminobenzidine was added as a visualizing agent. Nuclei were counterstained with hematoxylin. Positive staining for Col-1 and Col-3 was brown. Expression of Col-1 and Col-3 was compared between groups by calculating the ratio of positive staining area. A computer-aided morphometric analysis was used to quantitatively determine the positive staining area. Five fields of view were selected randomly from every slice, and the even integration optic density (IOD) value of the positive staining area was corrected by the blank in the same visual field.

### 4.8. Analysis of IL-6 and IL-17A mRNA Expression

Total RNA of lung tissue was isolated using Trizol reagent (Invitrogen, Carlsbad, CA, USA) according to manufacturer’s instructions. Two micrograms of total RNA from each sample were reverse-transcribed to cDNA which was used for PCR. GAPDH: sense GGGTGGTCCAGGGTTTCTTACT, antisense AGGTTGTCTCCTGCGACTTCA; IL-6: CAAAGCCAGAGTCCTTCAGAG, antisense GCCACTCCTTCTGTGACTCC; IL-17A: sense GCAAGAGATCCTGGTCCTGA, antisense AGCATCTTCTCGACCCTGAA. The optical density was recorded to evaluate the ratio of IL-6/GAPDH or IL-17A/GAPDH to determine the mRNA expression.

### 4.9. Western Blotting Analysis

Lung tissue samples were lysed by RIPA buffer containing 50 mM Tris-HCl (pH 7.2), 150 mM NaCl, 1% NP-40, 0.1% SDS, 1 mM EDTA, and 1 mM PMSF and homogenized in ice water for 30 min. After centrifugation (12,000 r/min, 10 min at 4 °C), the supernatant was collected, packed, and stored in −80 °C. Just before using, the loading buffer was mixed into the supernatant at a ratio of 1:4 and cooked in boiling water for 10 min. Then, the total protein concentration was determined by Bio-Rad Dc protein Assay Reagent (San Diego, CA, USA). Proteins in the supernatant were separated by SDS-PAGE on a 12% SDS polyacrylamide gel and then transferred to polyvinylidene fluoride (PVDF) membranes. The blotted membranes were blocked with 5% non-fat dairy milk (*w*/*v*). Blotted membranes were washed by 0.1% Tween-TBS (TBST) three times (10 min per time) after 2 h. Then blotted membranes were incubated at 4 °C overnight with primary anti- α -SMA antibodies (1:2000, Abcam, Cambridge, UK) and with secondary antibodies at room temperature for 1 h. After being washed with TBST three times (10 min per time), the blots were visualized with ECL reagent (Thermo Scientific, Rockford, IL, USA).

### 4.10. Statistical Analysis

The data are expressed as mean ± SD. Statistical testing was performed with SPSS software version 12.0 (SPSS Inc., Chicago, IL, USA). Comparison between groups was performed with test of homogeneity of variances and one-way analysis of variance (ANOVA), and *post hoc* analysis was performed using Bonferroni or Dunnett’s multiple comparison tests. Values of *p* < 0.05 and *p* < 0.01 were considered to be statistically significant.

## 5. Conclusions

This study demonstrated the anti-inflammatory properties of *m*KG and it also attenuated BLM-induced pulmonary fibrosis. Additionally, *m*KG showed some effects of regulating the ECM deposition and inhibiting the PF progression, and it may become an important method for preventing and treating PF.

## Supplementary Materials

Supplementary materials can be found at http://www.mdpi.com/1422-0067/17/2/238/s1.

## Abbreviations

PF: Pulmonary fibrosis
BLM: Bleomycin
*m*KG: Modified Gancao Kushen Formula
TGF-β: Transforming growth factor-β
HYP: Hydroxyproline
ROS: Reactive oxygen species
IL-17: Interleukin-17
IL-6: Interleukin-6
ECM: Extracellular matrix
α-SMA: α smooth muscle actin
SFE: Supercritical Fluid Extraction
*m*KG-L: Modified Gancao Kushen formula-Low dose
*m*KG-M: Modified Gancao Kushen formula-Middle dose
*m*KG-H: Modified Gancao Kushen formula-High dose
HE: Hematoxylin and eosin
PBS: Phosphate buffered saline
Col-1: Type 1 collagen
Col-3: Type 3 collagen
IOD: Integration optic density
ANOVA: One-way analysis of variance
EMT: Mesenchymal transition
