# Supplementary material for: The Chinese Herbal Medicine Formula mKG Suppresses Pulmonary Fibrosis of Mice Induced by Bleomycin
Source: Int J Mol Sci. 2016 Feb 15;17(2):238. doi: 10.3390/ijms17020238 (PMC4783969; doi:10.3390/ijms17020238)
Supplement: Supplementary file 1 [file ijms-17-00238-s001.pdf]

Supplementary Materials: The Chinese Herbal Medicine Formula *m*KG Suppresses Pulmonary Fibrosis of Mice Induced by Bleomycin

Ying Gao, Li-Fu Yao, Yang Zhao, Li-Man Wei, Peng Guo, Meng Yu, Bo Cao, Tan Li, Hong Chen and Zhong-Mei Zou

Quality Control of *m*KG Extract

The chemical constituents in *m*KG extract were profiled by UPLC-Q-TOF/MS[E] and GC-MS analysis (Figures S1 and S2). Major constituents were identified as matrine, oxymatrine, glycyrrhizic acid and ligustilide.

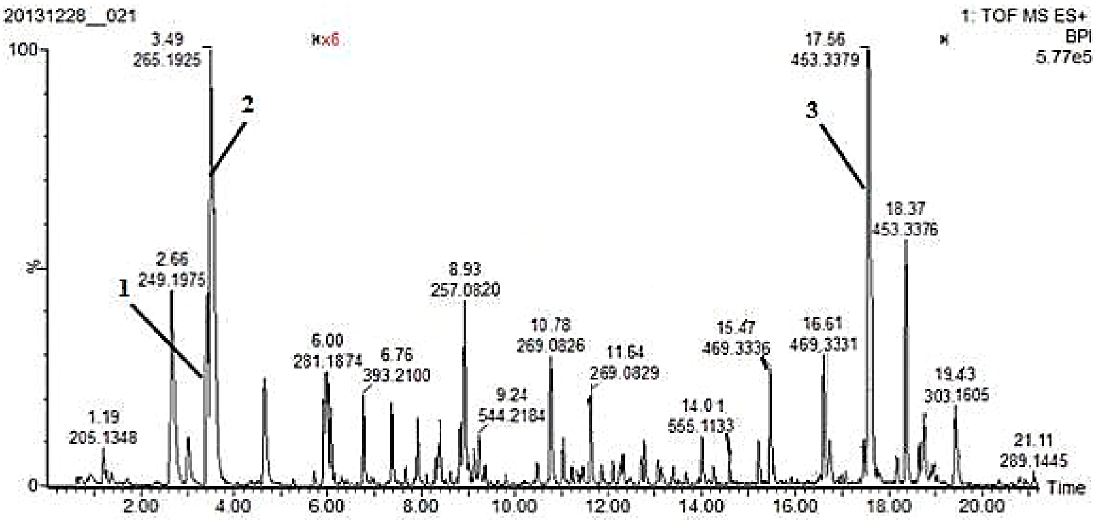

Figure S1. BPI chromatograms of *m*KG extract in positive ionization mode. 1: Matrine; 2: Oxymatrine; 3: Glycyrrhizic acid.

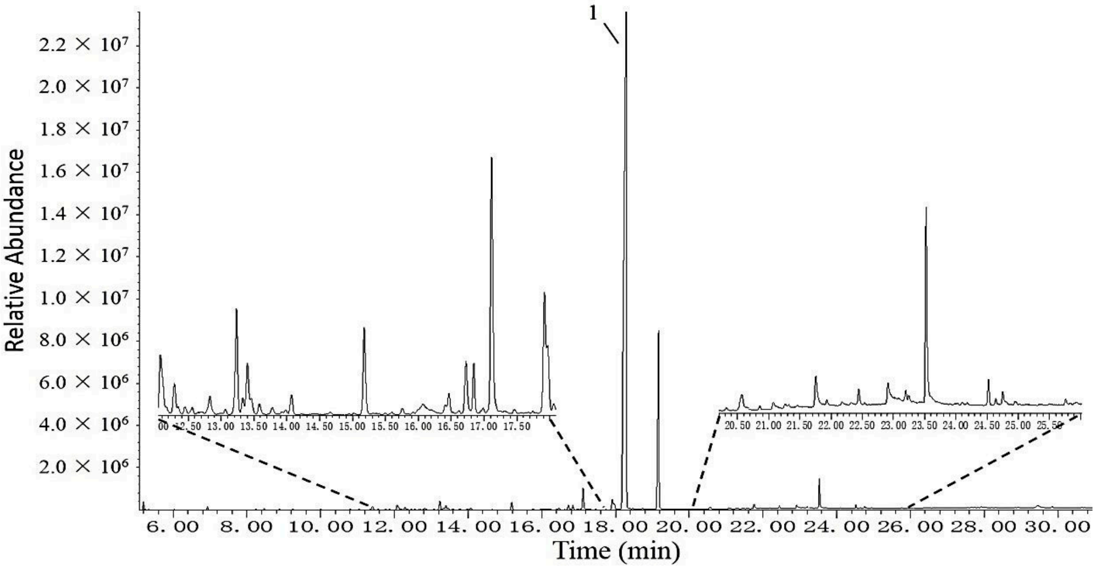

Figure S2. TIC chromatograms of Angelica oil analyzed by using the GC-MS. 1: Ligustilide.

UPLC-Q-TOF/MS Method. The *m*KG samples were analyzed on a Waters AcquityTM Ultra Performance LC system (Waters Corporation, Milford, MA, USA) equipped with a BEH C18 column (100 mm × 2.1 mm, 1.7 μm). The flow rate was 0.45 mL/min, the autosampler temperature was kept at 4 °C, the column compartment was set at 40 °C. The mobile phase was composed of water (A) and acetonitrile (B), each containing 0.1% formic acid. The gradient system for lung tissue samples was used: 0–0.5 min, 1% B; 0.5–5 min, 10% B; 5–13 min, 10%–50% B; 13–20 min, 50%–99% B; 20–22 min, washing with 99% B, and 22–25 min, equilibration with 1% B. The eluent from the column was directed to the mass spectrometer without split.

A Waters SYNAPT G2 HDMS (Waters Corp., Manchester, UK) was used to carry out the mass spectrometry with an electrospray ionization source (ESI) operating in positive ion mode. The capillary voltages were set at 3.0 KV, sample cone voltage 40 V, extraction cone voltage 4.0 V, respectively. Used drying gas nitrogen, the desolvation gas rate was set to 800 L/h at 400 °C, the cone gas rate at 50 L/h, and the source temperature at 100 °C. The scan time and inter-scan delay were set to 0.15 and 0.02 s, respectively. Leucine-enkephalin was used as the lockmass in all analyses (*m*/*z* 556.2771 for positive ion mode) at a concentration of 0.5 μg/mL with a flow rate of 5 μL/min. Data was collected in centroid mode from *m*/*z* 100 to *m*/*z* 1500.

GC-MS Method. The Angelica oil was analyzed by GC-MS using an Agilent computerized system 7890 gas chromatograph (Palo Alto, CA, USA), with an HP-5MS column (30 m × 0.25 mm i.d. × 0.25 μm film thickness) coated with 5% diphenyl and 95% dimethylpolysiloxane coupled to a 5977A mass spectrometer (Palo Alto, CA, USA). Helium carrier gas flowed at a rate of 1 mL/min, and injector and transfer line temperatures were 250 and 300 °C, respectively. The oven temperature was maintained at 60 °C for 5 min and then increased to 140 °C at a rate of 4 °C/min, then increased to 280 °C at a rate of 10 °C/min, at which temperature the column was maintained for 5 min. The split ratio was 1:10, and the mass spectrometer was operated in an electron ionization mode at 70 eV. The mass range scanned from *m*/*z* 50 to *m*/*z* 500 at 2.33 s/scan for a full-scan mode.
